# Arctic Psychrotolerant *Pseudomonas* sp. B14-6 Exhibits Temperature-Dependent Susceptibility to Aminoglycosides

**DOI:** 10.3390/antibiotics11081019

**Published:** 2022-07-29

**Authors:** Minjeong Kang, Tae-Rim Choi, Soyeon Ahn, Hee Young Heo, Hyerim Kim, Hye Soo Lee, Yoo Kyung Lee, Hwang-Soo Joo, Philip S. Yune, Wooseong Kim, Yung-Hun Yang

**Affiliations:** 1 College of Pharmacy, Graduate School of Pharmaceutical Sciences, Ewha Womans University, Seoul 03760, Korea; xov2038@ewhain.net (M.K.); sudht212@gmail.com (S.A.); hhy930@naver.com (H.Y.H.); gpfla9720@naver.com (H.K.); 2 Department of Biological Engineering, College of Engineering, Konkuk University, Seoul 05029, Korea; srim1004@gmail.com (T.-R.C.); lhs2265696@naver.com (H.S.L.); 3 Korea Polar Research Institute, Incheon 21990, Korea; yklee@kopri.re.kr; 4 Department of Biotechnology, College of Engineering, Duksung Women’s University, Seoul 01370, Korea; hwangsoojoo27@duksung.ac.kr; 5 Division of Infectious Diseases, Department of Medicine, Montefiore Medical Center, Albert Einstein College of Medicine, Bronx, NY 10467, USA; pyune@montefiore.org

**Keywords:** psychrotolerant bacteria, antibiotic tolerance, persisters, antibiotic resistance, aminoglycosides

## Abstract

Bacteria can evade antibiotics by acquiring resistance genes, as well as switching to a non-growing dormant state without accompanying genetic modification. Bacteria in this quiescent state are called persisters, and this non-inheritable ability to withstand multiple antibiotics is referred to as antibiotic tolerance. Although all bacteria are considered to be able to form antibiotic-tolerant persisters, the antibiotic tolerance of extremophilic bacteria is poorly understood. Previously, we identified the psychrotolerant bacterium *Pseudomonas* sp. B14-6 from the glacier foreland of Midtre Lovénbreen in High Arctic Svalbard. Herein, we investigated the resistance and tolerance of *Pseudomonas* sp. B14-6 against aminoglycosides at various temperatures. This bacterium was resistant to streptomycin and susceptible to apramycin, gentamicin, kanamycin, and tobramycin. The two putative aminoglycoside phosphotransferase genes *aph*1 and *aph*2 were the most likely contributors to streptomycin resistance. Notably, unlike the mesophilic *Pseudomonas* *aeruginosa* PA14, this cold-adapted bacterium demonstrated reduced susceptibility to all tested aminoglycosides in a temperature-dependent manner. *Pseudomonas* sp. B14-6 at a lower temperature formed the persister cells that shows tolerance to the 100-fold minimum inhibitory concentration (MIC) of gentamicin, as well as the partially tolerant cells that withstand 25-fold MIC gentamicin. The temperature-dependent gentamicin tolerance appears to result from reduced metabolic activity. Lastly, the partially tolerant *Pseudomonas* sp. B14-6 cells could slowly proliferate under the bactericidal concentrations of aminoglycosides. Our results demonstrate that *Pseudomonas* sp. B14-6 has a characteristic ability to form cells with a range of tolerance, which appears to be inversely proportional to its growth rate.

## 1. Introduction

Bacteria can acquire the ability to evade antibiotic activity by inhibiting intracellular penetration of antibiotics, effluxing antibiotics, modifying antibiotic targets, or inactivating antibiotics by horizontal gene transfer or de novo mutations [1]. Alternatively, bacteria can become transiently refractory to antibiotics by entering non-growing dormant states without obtaining these resistance mutations [2]. Bacteria in such a state are called persisters, which are characterized by high tolerance to multiple classes of antibiotics [2,3]. Most clinically utilized antibiotics work by inhibiting biosynthesis of nucleic acid, protein, and cell wall components required for bacterial growth [2]. Since these processes in dormant cells are significantly suppressed or even become inactive, antibiotics do not elicit bactericidal activity against persisters [2]. The bacterial persisters that exist inside biofilms confer high antibiotic tolerance to the sessile bacterial community [4]. Clinically, bacterial persisters are responsible for chronic and recurrent infections, including biofilm-related infections. There is no effective antimicrobial treatment to cure infections caused by persisters to date, and therefore it is imperative to understand the nature of antibiotic tolerance of persisters in order to adequately cope with the emerging antibiotic crisis.

Cold environments distant from human activity, such as permafrost, glaciers, and the Arctic and Antarctic soils, preserve microbial ecosystems that have remained practically unaffected by the anthropogenic use of antibiotics [5,6]. Investigations of antibiotic resistance in these pristine cold environments leads to a better understanding of the origins, evolution, and spread of antibiotic resistance [5,7,8]. For instance, it was previously considered that antibiotic-resistance was driven by anthropogenic misuse of antibiotics, since laboratory collections of bacteria isolated prior to clinical introduction of antibiotics had been reported to have no antibacterial resistance [9]. Several subsequent studies using the samples from the cold primeval biospheres proved that antibiotic resistance is an ancient natural phenomenon and the existence of antibiotic-resistance bacteria predates the discovery of antibiotics [5,7,10,11,12]. Following the confirmation of the existence of antimicrobial- or antifungal-producing bacteria in the cold biosphere [13,14,15], it has been widely posited that the existence of antibiotics in microbial communities may cause an evolutionary selective pressure on their vicinity, which consequently induces resistance via horizontal gene transfer or evolutionary mutations [7,16]. In addition, the identification of the gene (blaNDM-1) encoding New Delhi metallo-β-lactamase-1 protein from cold-adapted bacteria in High Arctic soil samples provided the evidence that antibiotic resistance can spread from humans to pristine environments [17].

Persister formation is widely accepted as a ubiquitous phenomenon found in all bacteria [18]. However, these findings were obtained based on mesophiles [3] and the antibiotic tolerance and persister formation of cold-adapted bacteria isolated from pristine environments remain largely elusive. In the present study, we sought to determine aminoglycoside resistance and tolerance of *Pseudomonas* sp. B14-6, a psychrotolerant bacterium isolated from a glacier foreland of the High Arctic. *Pseudomonas* sp. B14-6 had intrinsic resistance to streptomycin but was susceptible to other aminoglycosides, such as gentamicin, apramycin, and kanamycin, and tobramycin. Notably, we found that this bacterium showed increased tolerance to the susceptible aminoglycosides at low temperature, which appeared to result from reduced metabolic activity. We also provided experimental evidence that this cold-adapted bacterium could not only show partially tolerance to, but also concurrently proliferate in bactericidal concentrations of aminoglycosides without resistance mutations. To the best of our knowledge, this is the first report of the antibiotic tolerance of an Arctic psychrotolerant bacterium.

## 2. Results

### 2.1. Psychrotolerant Pseudomonas sp. B14-6 Shows Resistance to Streptomycin

**Figure 1:**
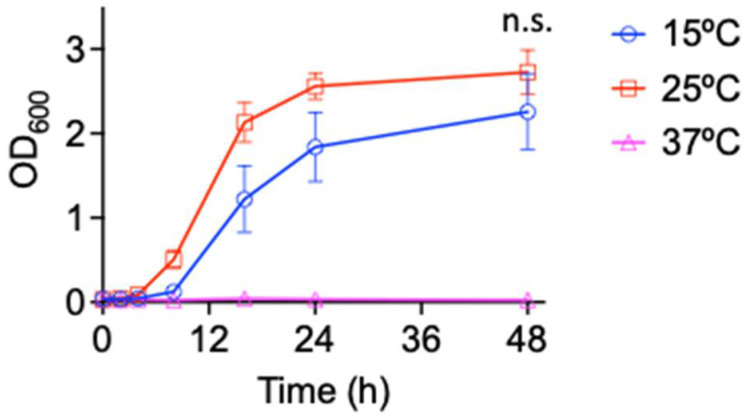
*Pseudomonas* sp. B14-6 is a psychrotolerant bacterium. *Pseudomonas* sp. B14-6 was grown at different temperature for 48 h. Bacterial growth was evaluated by absorbance measurement at 600 nm using a plate reader. Doubling times at 15 °C and 25 °C were 2.5 h and 1.7 h, respectively. Results are shown as mean ± SD; *n* = 3. OD600 between at 15 °C and 25 °C after 48-h incubation showed no significant difference (n.s. *p* > 0.05, *t*-test).

Bacteria that inhabit cold environments are generally categorized into psychrophiles, which grow optimally below 15 °C (upper limit of 20 °C), and psychrotolerant bacteria, which can survive at temperatures below 0 °C and grow optimally at 20–25 °C [19]. To define if *Pseudomonas* sp. B14-6 is either psychrotolerant or psychrophilic, a growth curve analysis at different temperature was performed. This species did not grow at 37 °C (Figure 1), whereas it grew to the stationary phase after 24 h of incubation at 25 °C (doubling time approximately 1.7 h), and after 48 h of incubation at 15 °C (doubling time approximately 2.5 h), respectively. This bacterium exhibited no statistically significant difference in final cell density at the stationary phase after 48-h incubation between 25 °C and 15 °C (*p* > 0.05).

To evaluate antibiotic-tolerance, bacteria need to be treated with a bactericidal antibiotic. Aminoglycosides, a class of antibiotics targeting bacterial ribosome, exhibit bactericidal activity against Gram-negative bacteria (including the *Pseudomonas* species) [20]. To determine if *Pseudomonas* sp. B14-6 is either susceptible or resistant to aminoglycosides, the minimum inhibitory concentrations (MICs) of a panel of aminoglycosides were measured at the optimal temperature of 25 °C. As shown in Table 1, *Pseudomonas* sp. B14-6 was susceptible to gentamicin (MIC 0.5 µg/mL), tobramycin (MIC 0.5 µg/mL), kanamycin (MIC 2 µg/mL), and apramycin (MIC 4 µg/mL) at 25 °C. However, this bacterium was resistant to streptomycin (MIC 32 µg/mL), which was comparable to the mesophilic *Pseudomonas aeruginosa*, often displays resistance to streptomycin (Table 1) [21,22]. Taken together, these results indicate that *Pseudomonas* sp. B14-6 is categorized as a psychrotolerant organism. Remarkably, this particular organism displays a characteristic resistance to streptomycin, without demonstrating a global resistance pattern to other aminoglycosides.

### 2.2. Identification of the Determinants of Aminoglycoside Resistance

To further investigate streptomycin resistance, we analyzed whole-genome sequencing data of *Pseudomonas* sp. B14-6 (Genbank accession number CP053929) [23] and identified determinants of antibiotic resistance by conducting a BLAST search against CARD (https://card.mcmaster.ca, accessed on 20 April 2021) [24,25]. As shown in Table S1, dozens of antibiotic resistance determinants against major classes of antibiotics, such as beta-lactams, fluoroquinolones, macrolides, and aminoglycosides, were present in *Pseudomonas* sp. B14-6. Moreover, multiple genes encoding various antibiotic efflux pumps, such as the major facilitator superfamily (MFS), the resistance-nodulation cell division (RND) family, and the ATP-binding cassette (ABC) superfamily, were detected in the genome of this bacterium (Table S1). Aminoglycoside-resistance in *Pseudomonas* species is achieved by producing aminoglycoside-modifying enzymes, reducing intracellular aminoglycoside accumulation, or altering ribosomal targets [20]. While the decrease in the cellular uptake of aminoglycosides or modification of ribosomal targets tends to confer resistance to a wide range of aminoglycosides, inactivation of aminoglycosides *per se* by enzymes generally confers resistance to specific aminoglycosides [26].

Indeed, the *Pseudomonas* sp. B14-6 genome contained two putative aminoglycoside phosphotransferases (APHs) (PubMed accession no. WP_172791649.1 and WP_145309966.1, designated respectively as *aph1* and *aph2* throughout this article) (Table S1). The BLAST results revealed that *aph1* and *aph2* were homologous to genes encoding APH(3′) and APH(2″) among the APH family, respectively (Table 2). Furthermore, *aph1* showed 99% homology to bifunctional aminoglycoside phosphotransferase/ATP-binding protein from *Pseudomonas mandelii* (NCBI Reference Sequence: WP_010467625.1); *aph2* exhibited 99% homology to aminoglycoside phosphotransferase from *Pseudomonas mandelii* JR-1 (NCBI Reference Sequence: AHZ71124.1). Although other aminoglycoside-modifying enzymes, such as aminoglycosides acetyltransferases or aminoglycoside nucleotidyltransferases, can also modify aminoglycosides [27], no genes encoding these enzymes have been found in the *Pseudomonas* sp. B14-6 genome.

**Figure 2:**
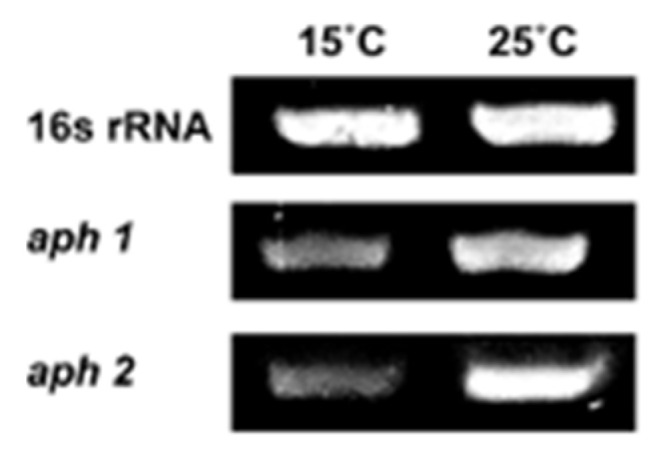
*aph*1 and *aph*2 are transcribed at 15 °C or 25 °C. The mRNA levels of *aph*1 and *aph*2 were quantified by semi-quantitative RT-PCR. Total RNA was extracted from *Pseudomonas* sp. B14-6 grown for 24 h at 15 °C and 25 °C, respectively. 16S rRNA was used as a loading control.

Although antibiotic resistance genes exist in bacteria, they are often unexpressed [28,29]. We verified the expression of *aph1* and *aph2* by RT-PCR. As shown in Figure 2, both *aph1* and *aph2* were transcribed at 15 °C and 25 °C, indicating that they are not silenced in *Pseudomonas* sp. B14-6. Combined with the MIC results (Table 1), it is most likely the case that *aph1* or *aph2* are responsible for streptomycin resistance of *Pseudomonas* sp. B14-6.

### 2.3. Pseudomonas sp. B14-6 Shows Reduced Susceptibility to Aminoglycosides at Low Temperature

The Gram-negative model organism *Escherichia coli* grown at low temperatures is known to become more susceptible to antibiotics [30]. We also observed the similar phenomenon in the mesophilic *Pseudomonas aeruginosa* strain PA14 (Table 1). As temperature was reduced from 37 °C to 15 °C, MICs of both gentamicin against *P. aeruginosa* PA14 decreased 8-fold from 2 µg/mL to 0.25 µg/mL (Table 1). Tobramycin and apramycin behaved similarly as their MICs reduced from 0.5 µg/mL to 0.125 µg/mL, and from 8 µg/mL to 2 µg/mL, respectively, by reducing the incubation temperature from 37 °C to 15 °C (Table 1). More notably, MIC of streptomycin substantially deceased from 64 µg/mL at 37 °C to 2 µg/mL at 15 °C (Table 1). While *P. aeruginosa* PA14 was not susceptible to kanamycin (MIC > 64 µg/mL) at 37 °C, it also showed the MIC of 64 µg/mL at 15 °C (Table 1). Interestingly, however, MICs of these aminoglycosides against *Pseudomonas* sp. B14-6 did not change or increased 2-fold as growth temperature decreased from 25 °C to 15 °C (Table 1). These results indicate that unlike mesophilic *P. aeruginosa*, the psychrotolerant *Pseudomonas* sp. B14-6 exhibited a low temperature-induced decrease in aminoglycoside susceptibility. Next, we tested if this low-temperature induced decrease in susceptibility was due to the acquisition of resistance mutation by determining the MICs of aminoglycosides against the progenies of the 15 °C-grown cells at 25 °C. These progenies exhibited no increase in MICs to aminoglycosides. The MICs of gentamicin, tobramycin, apramycin, and kanamycin were still, 0.5 µg/mL, 0.5 µg/mL, 2 µg/mL, and 4 µg/mL, respectively at 25 °C (Table 1). These results indicate that this susceptibility change in a low temperature might be related to an increase in antibiotic-tolerance rather than resistance.

### 2.4. Pseudomonas sp. B14-6 Forms a Small Portion of Persisters Only at a Low Temperature

**Figure 3:**
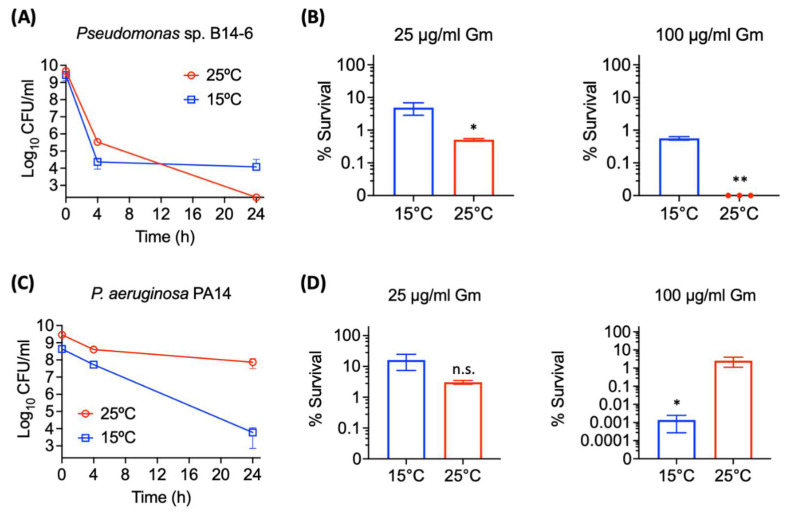
Aminoglycoside-tolerant population of *Pseudomonas* sp. B14-6 is composed of partially tolerant cells and high tolerant persisters. The overnight cultures of *Pseudomonas* sp. B14-6 (**A**) or of *P. aeruginosa* PA14 (**C**) were treated with or 100 µg/mL gentamicin for 24 h at 15 °C and 25 °C. The dependent viability was measured at 4 h and 24 h post treatment. *Pseudomonas* sp. B14-6 overnight culture (**B**) or *P. aeruginosa* PA14 overnight culture (**D**) were treated with 25 µg/mL gentamicin, or 100 µg/mL gentamicin for 24 h at 15 °C and 25 °C. Viability was measured by serial dilution and plating on agar plates. The data points on the x-axis (**B**) are below the level of detection (2 × 10^2^ CFU/ml). Results are shown as mean ± SD; *n* = 3. Statistical differences were analyzed by *t*-test; * *p* < 0.05, ** *p* < 0.01, n.s. not significant (*p* > 0.05).

The extent of aminoglycoside tolerance of *Pseudomonas* sp. B14-6 upon different temperatures was evaluated. As a first step, its ability to form bacterial persisters was assessed. It is recognized that one of the distinctive features of persistence is the biphasic killing curve after the exposure to a high concentration of bactericidal antibiotics [2]. To evaluate persistence in *Pseudomonas* sp. B14-6, overnight cultures of *Pseudomonas* sp. B14-6 were treated with 100 µg/mL gentamicin for 24 h at 25 °C and 15 °C, respectively. *Pseudomonas* sp. B14-6 exhibited biphasic killing patterns at both temperatures. Culturing at 15 °C left with a subpopulation of about 4.5-log CFU/mL persisters that were neither dying nor growing cells, while the cells were killed at 25 °C, albeit at a slower rate after 4 h. No persisters were observed in this population (Figure 3A). When the remaining survival cells grew in fresh media, the progeny was susceptible to gentamicin (MIC 0.5 µg/mL at 25 °C), indicating that the survivals were not resistant cells.

Bacterial populations under antibiotic stress may consist of the susceptible, partially tolerant, and high tolerant persister subpopulations [31]. In particular, the bacterial subset showing slow-killing kinetic is recognized to have partial tolerance, where the extent of tolerance is higher than the susceptible cells, but lower than the persister cells [31]. It was hypothesized that the slowly killed bacterial population observed at 25 °C was the partially tolerant subset. The size of the antibiotic-tolerant subpopulation surviving after 24-h exposure to 25 µg/mL and 100 µg/mL gentamicin was estimated at 15 °C and at 25 °C, respectively. After the treatment with 25 µg/mL gentamicin, 5.4% of the cell population incubated at 15 °C exhibited tolerance, while 0.5% of the population was tolerant to gentamicin at 25 °C (Figure 3B). Similarly, 0.5% of the cell population was the persister cells tolerant to 100 µg/mL gentamicin at 15 °C. In contrast, no *Pseudomonas* sp. B14-6 cells survived in such high concentration of gentamicin at 25 °C (Figure 3B), indicating that the remaining viable cells under 25 µg/mL gentamicin were neither persisters nor resistant cells. These results indicate that *Pseudomonas* sp. B14-6 can form aminoglycoside-tolerant cells, consisting of a large portion of partially tolerant cells and a small portion of persisters showing high tolerance to aminoglycosides. Furthermore, the overall percentage of aminoglycoside-tolerant cells increases as temperature decreases. In contrast to *Pseudomonas* sp. B14-6, the mesophilic *P. aeruginosa* PA14 formed larger amount of persisters at 25 °C than at 15 °C, indicating that low temperature-induced increase in antibiotic tolerance is a unique feature of *Pseudomonas* sp. B14-6.

### 2.5. Low Metabolic Activity Is Correlated with Aminoglycoside Tolerance

**Figure 4:**
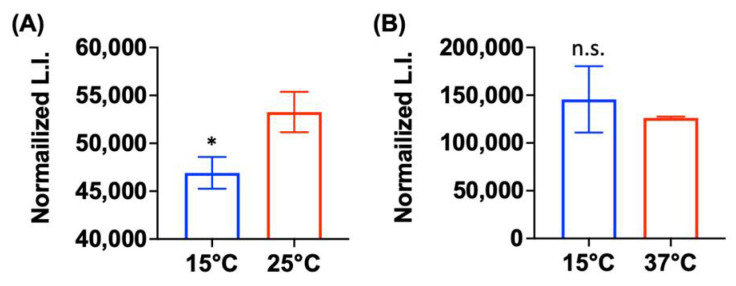
Psychrotolerant *Pseudomonas* sp. B14-6 displayed lower ATP levels at 15 °C vs. 25 °C. Intracellular ATP levels in *Pseudomonas* sp. B14-6 (**A**) or *P*. *aeruginosa* PA14 (**B**) were estimated by the luciferase reaction in lysed cell pellets. Luminescence intensity (L.I.) was normalized by cell concentration. Results are shown as mean ± SD; *n* = 3. Statistical differences were analyzed by *t*-test; * *p* < 0.05, n.s. not significant (*p* > 0.05).

It is widely accepted that antibiotic tolerance of bacteria is altered by metabolic state, in which ATP levels can serve as a marker [32,33]. Based on this concept, we hypothesized that the temperature-dependent increase in aminoglycoside tolerance of *Pseudomonas* sp. B14-6 might result from the alteration in its metabolic activity. To test this hypothesis, we assessed the metabolic activity by measuring cellular ATP levels. As shown in Figure 4, *Pseudomonas* sp. B14-6 grown at 15 °C exhibited significantly reduced ATP levels compared to the levels at 25 °C (*p* < 0.05). However, the ATP levels inside *P. aeruginosa* PA14 was not significantly different between 37 °C and 15 °C (Figure 4). This result indicates psychrotolerant *Pseudomonas* sp. B14-6 grown at 15 °C is in a lower metabolic state compared to at 25 °C. Taken together, its aminoglycoside tolerance at low temperature is mostly likely attributed to the reduced metabolic activity.

### 2.6. Pseudomonas sp. B14-6 Can Slowly Proliferate under Bactericidal Concentrations of Aminoglycoside

**Figure 5:**
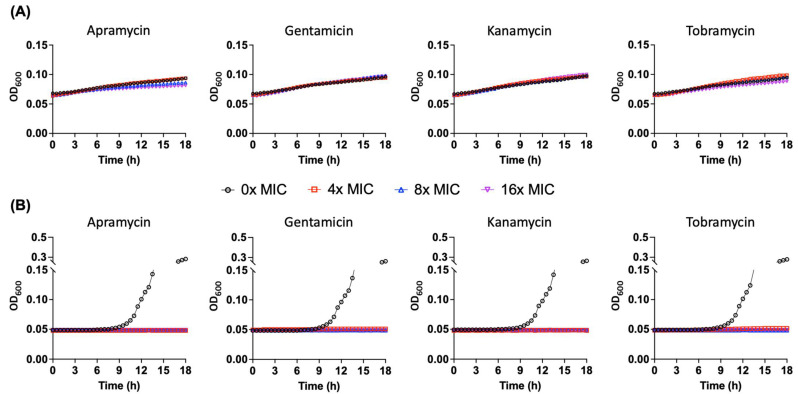
Survivors of *Pseudomonas* sp. B14-6 after treatment with gentamicin are capable of slowly replicating in bactericidal concentrations of aminoglycosides. *Pseudomonas* sp. B14-6 overnight culture was treated with 25 µg/mL gentamicin (**A**) or non-treated (**B**) for 18 h at 25 °C. After washing, ~10^4^ CFU/mL of remaining cells were treated with the indicated concentrations of aminoglycosides for 24 h at 25 °C. Bacterial growth was evaluated by absorbance measurement at 600 nm using a plate reader. Results are shown as mean (*n* = 3). The error bars are not shown for clarity.

Unlike persisters, partially tolerant cells have an ability to replicate under antimicrobial stress [31]. To elucidate that the partially tolerant *Pseudomonas* sp. B14-6 could proliferate in presence of susceptible aminoglycosides, the survivor cells after exposure to 25 µg/mL gentamicin for 24 h at 25 °C were procured. These cells were incubated with a range of concentrations of aminoglycosides for additional 18 h at 25 °C (Figure 5A). A control group of *Pseudomonas* sp. B14-6, which was not pre-treated with gentamicin, was also treated with comparable concentrations of aminoglycosides at the same growth temperature (Figure 5B). The non-gentamicin-treated *Pseudomonas* sp. B14-6 cells showed normal growth with the doubling time of 1.7–1.9 h after an approximately 7-h lag phase in the absence of aminoglycosides (Figure 5B and Table S2). However, these non-treated cells did not grow under equal or higher than 4× MIC (i.e., defined as the minimum bactericidal concentration) of apramycin, gentamicin, kanamycin, and tobramycin (Figure 5B and Table S2). Remarkably, the remaining survivor cells after exposure to 25 µg/mL gentamicin did slowly grow regardless of the presence of aminoglycosides from 0 to 16× MICs with the doubling time of 26.5–55.0 h (Figure 5A and Table S2). It should be noted that the progeny of these partially tolerant cells obtained after the completion of the 18-h incubation time was still susceptible to apramycin, gentamicin, kanamycin, and tobramycin with MICs of 4 µg/mL, 0.5 µg/mL, 2 µg/mL, 0.5 µg/mL, respectively, at 25 °C (Table 1), suggesting that the partial tolerance did not carry on to the progeny. These results illustrate that the psychrotolerant *Pseudomonas* sp. B14-6 formed the partially tolerant cells which are capable of replicating in the presence of bactericidal antibiotics without acquiring resistant mutations.

## 3. Discussion

Antibiotic tolerant cells are stochastically generated from normal susceptible cells, and their antibiotic tolerance is known to strongly correlate with growth rates and ATP levels [32,34,35,36,37]. Since the rate of biosynthetic processes are reduced in slow-growing or metabolically inactive bacteria, most antibiotics are rendered less effective against these antibiotic-tolerant bacteria owing to the lack of active targets [38,39,40]. Antibiotic tolerance is maximized once bacterial growth ceases [34]. Reversible transition to a low metabolic state, defined as dormancy, is a universal strategy employed by a wide range of microorganisms against unfavorable stressors [41,42]. For instance, despite differences in the types of stress between environmental and clinical conditions, a common response to hostile conditions is to arrest growth [43].

Claudi et al. reported that bacterial population under antimicrobial stress was composed of the subpopulations with a diverse antibiotic-tolerance, such as susceptible cells, partially tolerant cells, and highly tolerant persister cells [31]. Their study demonstrated that while fast-growing cells were quickly eradicated by antibiotic treatment, non-growing, moderate-growing, and slow-growing cells survived. Furthermore, among the surviving cells, non-growing cells were rare, but moderate-and slow-growing cells exhibiting partial tolerance were abundant [31]. *Salmonella* in dendritic cells and *Mycobacteria* in macrophages can also divide slowly while being tolerant to antibiotics [44,45]. The phenotype of these partially tolerant replicating bacterial cells has been observed only *in cellulo* but not in vitro. In the present study, *Pseudomonas* sp. B14-6 clearly showed this phenotype in LB broth culture, with its growth and tolerance detected by simple OD measurement (Figure 5A). After treating the cells with bactericidal concentrations of aminoglycosides, most surviving cells were partially tolerant cells rather than non-growing persisters. These results suggest that the psychrotolerant *Pseudomonas* sp. B14-6 may have a similar strategy of antibiotic tolerance as that adopted by intracellular pathogens.

The molecular mechanisms underlying the growth of *Pseudomonas* sp. B14-6 in the presence of lethal concentrations of aminoglycosides without acquiring resistance remain elusive. Given that protein synthesis still occurs in persisters [46], and potentiation of aminoglycoside uptake leads to the killing of bacterial persisters [47,48,49,50], it is thought that the tolerance to antibiotics may result from low intracellular concentrations of aminoglycosides, and less likely from inactivation of drug targets by the organisms. This argument is supported by demonstrating that lowering incubation temperature resulted in an increased tolerance to aminoglycosides (Figure 3A,B) and that is also resulted in a concomitant decrease in metabolic activity (Figure 4A). The concentration of intracellular aminoglycosides can be lowered by inhibiting their uptake. Since metabolic energy generating systems promote proton motive force [51], the decreased metabolic activity at low temperature (Figure 4A) could retard cellular uptake of aminoglycosides in *Pseudomonas* sp. B14-6.

In conclusion, the psychrotolerant bacterium *Pseudomonas* sp. B14-6 exhibited temperature-dependent tolerance to aminoglycosides by modulating its metabolic activity. Particularly, *Pseudomonas* sp. B14-6 formed the slowly replicating cells that were partially tolerant to aminoglycosides. These results verify that bacteria can display antibiotic tolerance while simultaneously replicating in the presence of lethal levels of antibiotics without acquiring resistance. Considering that slow-growing and moderate-growing bacteria play a crucial role in recurrent chronic infections, our findings provide an additional insight in understanding bacterial strategies to survive physiologic stress imposed by antibiotics.

## 4. Materials and Methods

### 4.1. Bacterial Strain and Growth Conditions

*Pseudomonas* sp. B14-6 was previously isolated from the glacier foreland soil of Midtre Lovénbreen in High Arctic Svalbard (78.9° N, 12.0° E) [23]. To isolate individual colonies, the frozen stock of *Pseudomonas* sp. B14-6 was streaked on a Luria-Bertani (LB) agar plate and incubated at 30 °C overnight. To prepare the overnight culture, a single colony of *Pseudomonas* sp. B14-6 was inoculated into 5 mL Luria-Bertani (LB; BD Cat. No. 244620) broth and incubated at 30 °C with shaking (200 rpm) for 24 h. Since this bacterium grew at 4 °C, which caused merging of the colonies, the frozen stock was freshly streaked for preparing the overnight culture. The mesophilic *P*. *aeruginosa* PA14 [52] was grown in LB broth (BD, Franklin Lakes, NJ, USA) at 37 °C at 200 rpm.

### 4.2. Chemicals

Apramycin, gentamicin, kanamycin, streptomycin, and tobramycin were purchased from Sigma-Aldrich (St. Louis, MO, USA). Stock solutions (10 mg/mL) of all antibiotics were prepared in double-distilled water (ddH_2_O).

### 4.3. Determination of Minimum Inhibitory Concentrations (MICs)

MIC values were evaluated by the microbroth dilution method as proposed by Clinical & Laboratory Standards Institute (CLSI) guidelines [53]. Cation-adjusted Mueller Hinton (CaMH) broth (BD Cat. No. 212322) was used as the assay medium for MIC determination. Bacterial growth was determined based on the optical density at 600 nm using a Cytation 5 multi-mode reader (BioTek, Winooski, VT, USA) or an Infinite M200 Pro microplate reader (Tecan Group Ltd., Männedorf, Switzerland) equipped at Ewha Drug Development Research Core Center. MIC values against *Pseudomonas* sp. B14-6 were measured after 48 h incubation at 15 °C or after 24 h incubation at 25 °C. MICs against *P. aeruginosa* PA14 were evaluated after 72 h incubation at 15 °C, after 48 h incubation at 25 °C, or after 24 h incubation at 37 °C. Bacterial growth was defined as OD600 nm ≥ 0.1. The assay was conducted in biological triplicate.

### 4.4. Identification of Antibiotic Resistance Genes

Whole-genome sequencing of *Pseudomonas* sp. B14-6 has been previously completed and deposited in Genbank (accession number CP053929) and in BioProject (ID PRJNA634457) [23]. The genomes consisting of 6,776,772 base pairs were annotated by KEGG Automatic Annotation Server (KAAS, https://www.genome.jp/kegg/kaas, accessed on 20 April 2021) [54] to identify antibiotic resistance genes. Subsequently, the BLASTp analysis of the identified putative antibiotic resistant genes was conducted against the Comprehensive Antibiotic Resistance Database (CARD, https://card.mcmaster.ca, accessed on 20 April 2021) [24,25].

### 4.5. Reverse Transcription-Polymerase Chain Reaction (RT-PCR)

*Pseudomonas* sp. B14-6 was cultured in LB medium at 15 °C or 25 °C with shaking (200 rpm) for 48 h. Total RNA was extracted by using the RNeasy Mini Kit (Qiagen, Hilden, Germany) following the manufacturer’s protocol. The NanoDrop system (Thermo Fisher Scientific, Waltham, MA, USA) was used for determining RNA purity and quantity. Samples were diluted with DEPC-treated water (RNase free) to normalize the RNA concentration in samples. First-strand cDNA was synthesized using random hexamer primers of Invitrogen SuperScript III kit (Carlsbad, CA, USA) following the manufacturer’s protocol. 16S rRNA gene was used as a control in the semi-quantitative RT-PCR analysis. The concentrations of PCR products were compared after 1% agarose gel electrophoresis.

### 4.6. Determination of Aminoglycoside-Tolerant Cells

The overnight culture was diluted 1:200 in 25 mL LB broth and incubated at 15 °C or 25 °C for 24 h with shaking (200 rpm). Aliquots (1 mL) of each culture were transferred to a 96-well assay block (Corning Costar 3960; Corning, NY, USA), to which the desired concentrations of aminoglycosides were added. The assay bock was then incubated at 15 °C or 25 °C for an additional 24 h with shaking (200 rpm). At the indicated time after incubation, 100 µL aliquots were serially diluted with phosphate-buffered saline (PBS) and spot-plated on CaMH agar plates to enumerate the number of viable cells. These experiments were conducted in biological triplicate.

### 4.7. Comparison of Intracellular ATP Levels

The ATP level was determined by using the BacTiter-GloTM Microbial Cell Viability Assay kit (Promega Corporation, Cat # G8231, Dübendorf, Switzerland) following the manufacturer’s instruction as previously described [55]. Briefly, the overnight culture was diluted 1:1000 in 5 mL LB broth and then incubated at 15 °C, 25 °C, or 37 °C with shaking (200 rpm) for 24 h. After incubation, the bacterial cultures grown at each temperature were adjusted to the same concentration. Next, 50 µL of the culture samples was dispensed into a black, clear-bottom, 96-well plate (Corning Costar 3603) and then mixed with the same volume of BacTiter-GloTM reagent. After 10 min, the luminescence was measured using a Cytation 5 multi-mode reader (BioTek). The assay was conducted in biological triplicate. Statistical significance was tested by the paired *t*-test using GraphPad Prism 8 (GraphPad Software, La Jolla, CA, USA). A *p*-value of less than 0.05 was considered to indicate statistical significance.

### 4.8. Indentification of Partially Tolerant Slow Growing Cells

The overnight culture of *Pseudomonas* sp. B14-6 was diluted 1:200 in 25 mL LB broth and incubated at 25 °C with shaking (200 rpm) for 24 h in 250 mL flask. The bacterial culture was then treated with 25 µg/mL gentamicin at 25 °C and 200 rpm for 24 h. For negative control group, the treatment with gentamicin was omitted. The remaining cells after treatment were washed, and their concentrations were adjusted to ~2 × 10^4^ CFU/mL in LB broth. Fifty µg/mL of the cell suspension was added to each well of the 96-well plate filled with 50 µg/mL LB broth including a range of concentrations of aminoglycosides. One hundred µg/mL of mineral oil (Sigma Cat. No. M5904) was added to each well to prevent evaporation. The assay plate was incubated at 25 °C for 18 h in a Cytation 5 multi-mode reader (BioTek), and the OD600 was measured every 30 min. A log plot depicting OD600 as a function of time was drawn for each set of experiment. The exponential growth phase showed a linear relationship between log OD600 versus time. Linear regression was performed utilizing Excel (Microsoft Corporation, Redmond, WA, USA) to derive the formula of the best fitting line in linear function, where the slope is defined as the growth rate (R-squared coefficient > 0.9). The doubling time was calculated by dividing ln 2 by the growth rate. The experiment was conducted in triplicate.

## Figures and Tables

**Table 1:** Minimum inhibitory concentration (µg/mL) at different temperature.

	*Pseudomonas* sp. B14-6		*P. aeruginosa* PA14		
Antibiotics	15 °C	25 °C	15 °C	25 °C	37 °C
Gentamicin	1	0.5	0.25	2	2
Tobramycin	0.5	0.5	0.125	0.5	0.5
Apramycin	8	4	2	8	8
Kanamycin	2	2	64	>64	>64
Streptomycin	64	32	2	16	32

**Table 2:** List of antibiotic resistance-related genes in *Pseudomonas* sp. B14-6.

Sequence Name	Sequence Description	Sequence Length	Gene Ontologies
orf04666	aminoglycoside phosphotransferase (*aph1*)	1557	P:metabolic process; F:transferase activity, transferring phosphorus-containing groups
orf05258	aminoglycoside phosphotransferase (*aph2*)	843	P:metabolic process; F:transferase activity, transferring phosphorus-containing groups

## Data Availability

The datasets generated during and/or analyzed during the current study are available from the corresponding author on reasonable request.

## Supplementary Materials

The following supporting information can be downloaded at: https://www.mdpi.com/article/10.3390/antibiotics11081019/s1, Table S1: Antimicrobial resistance determinants found in *Pseudomonas* sp. B14-6; Table S2: Doubling time (hour) of *Pseudomonas* sp. B14–6.
