# Supplementary material for: Arctic Psychrotolerant Pseudomonas sp. B14-6 Exhibits Temperature-Dependent Susceptibility to Aminoglycosides
Source: Antibiotics (Basel). 2022 Jul 29;11(8):1019. doi: 10.3390/antibiotics11081019 (PMC9405152; doi:10.3390/antibiotics11081019)
Supplement: Supplementary file 1 [file antibiotics-11-01019-s001.zip › antibiotics-1753697-SI.pdf]

Supplementary Information

Table S1. Antimicrobial resistance determinants found in *Pseudomonas* sp. B14-6

**PubMed accession no.**	**CARD Blast results**			
	**Name**	**E-value**	**AMR Gene Family**	**Drug Class**
WP_172790574.1	PDC-41	8.86e-167	PDC beta-lactamase	carbapenem, cephalosporin, monobactam
WP_172790875.1	RSA-2	9.68	RSA beta-lactamase	carbapenem, cephalosporin
WP_172790876.1	*arnA*	0	pmr phosphoethanolamine transferase	peptide antibiotic
WP_172790877.1	PmrF	1.30e-158	pmr phosphoethanolamine transferase	peptide antibiotic
WP_172791052.1	*bacA*	5.43e-70	undecaprenyl pyrophosphate related proteins	peptide antibiotic
WP_077747783.1	EreB	6.95e-11	macrolide esterase	Macrolide
WP_172791079.1	*clbB*	0.91	Cfr 23S ribosomal RNA methyltransferase	streptogramin, lincosamide, phenicol, oxazolidinone, pleuromutilin
WP_077748253.1	*bacA*	1.49e-71	undecaprenyl pyrophosphate related proteins	Peptide antibiotic
WP_154501557.1	*Neisseria gonorrhoeae* PBP1 conferring resistance to beta-lactam	0.94	penicillin-binding protein mutations conferring resistance to beta-lactam antibiotics	penam, cephamycin, cephalosporin
WP_169857918.1	*Neisseria meningititis* PBP2 conferring resistance to beta-lactam	3.11e-120	penicillin-binding protein mutations conferring resistance to beta-lactam antibiotics	cephalosporin, penam, cephamycin
WP_077748995.1	*Staphylococcus aureus gyrA* conferring	1.34	fluoroquinolone resistant *gyrA*	fluoroquinolone

	resistance to fluoroquinolones			
WP_077750439.1	*Streptococcus pneumoniae* PBP1a conferring resistance to amoxicillin	4.83e-53	penicillin-binding protein mutations conferring resistance to beta-lactam antibiotics	penam, cephalosporin, cephamycin
WP_172791649.1	APH(3')-Ib	0.11	APH(3')	aminoglycoside
WP_010467364.1	*Neisseria meningitis* PBP2 conferring resistance to beta-lactam	7.92e-47	penicillin-binding protein mutations conferring resistance to beta-lactam antibiotics	cephalosporin, penam, cephamycin
WP_145309966.1	APH(2'')-Ig	0.04	APH(2'')	Aminoglycoside
WP_172791890.1	OpmD	5.87	resistance-nodulation-cell division (RND) antibiotic efflux pump	disinfecting agents and intercalating dyes, acridine dye, tetracycline, fluoroquinolone
WP_002555808.1	LpsA	4.03	intrinsic peptide antibiotic resistant Lps	peptide antibiotic
WP_172792052.1	*Pseudomonas aeruginosa gyrA* and *parC* conferring resistance to fluoroquinolones	0.47	fluoroquinolone resistant parC	fluoroquinolone
WP_172792062.1	*Neisseria gonorrhoeae* PBP1 conferring resistance to beta-lactam	0	penicillin-binding protein mutations conferring resistance to beta-lactam antibiotics	penam, cephalosporin, cephamycin
WP_010463662.1	TEM-198	0.04	TEM beta-lactamase	penam, cephalosporin, penem, monobactam
WP_172792348.1	*Staphylococcus aureus* norA	0.26	major facilitator superfamily (MFS) antibiotic efflux pump	fluoroquinolone

WP_010458391.1	*Neisseria meningititis* PBP2 conferring resistance to beta-lactam	9.16e-49	penicillin-binding protein mutations conferring resistance to beta-lactam antibiotics	cephalosporin, penam, cephamycin
WP_172792493.1	OprZ	3.23	resistance-nodulation-cell division (RND) antibiotic efflux pump	macrolide, fluoroquinolone, cephalosporin, aminoglycoside
WP_169857413.1	*macB*	1.94e-164	ATP-binding cassette (ABC) antibiotic efflux pump	Macrolide
WP_172790691.1	*dfrA10*	5.14	trimethoprim resistant dihydrofolate reductase dfr	diaminopyrimidine
WP_010459887.1	*vanL*	3.29	glycopeptide resistance gene cluster, van ligase	Glycopeptide
WP_172791504.2	*mphN*	0.75	macrolide phosphotransferase (MPH)	Macrolide
WP_077750448.1	LRA-9	6.03	subclass B3 LRA beta-lactamase	cephalosporin, penam
WP_010463795.1	*Enterococcus faecium* cls conferring resistance to daptomycin	5.59	daptomycin resistant cls	peptide antibiotic
WP_169856493.1	*Enterococcus faecalis* liaR mutant conferring daptomycin resistance	0.79	daptomycin resistant liaR	peptide antibiotic
WP_154503919.1	*carA*	0.05	ABC-F ATP-binding cassette ribosomal protection protein	phenicol, oxazolidinone, streptogramin, lincosamide, tetracycline, macrolide, pleuromutilin
WP_172790599.1	*mdtP*	1.36e-53	major facilitator superfamily (MFS) antibiotic efflux pump	disinfecting agents and intercalating dyes, nucleoside antibiotic, acridine dye

WP_172790600.1	*emrA*	2.21e-105	major facilitator superfamily (MFS) antibiotic efflux pump	fluoroquinolone
NZ_CP053929.1 (791,226..792,541)	MuxA	4.77e-136	resistance-nodulation-cell division (RND) antibiotic efflux pump	macrolide, tetracycline, aminocoumarin, monobactam
NZ_CP053929.1 (791,226..792,541)	Tet(X1)	1.45	tetracycline inactivation enzyme	Tetracycline
WP_010459912.1	MuxB	0	resistance-nodulation-cell division (RND) antibiotic efflux pump	tetracycline, monobactam, macrolide, aminocoumarin
WP_172790857.1	*adeB*	0	resistance-nodulation-cell division (RND) antibiotic efflux pump	tetracycline, glycylcycline
WP_169857770.1	*cmeB*	0	resistance-nodulation-cell division (RND) antibiotic efflux pump	macrolide, cephalosporin, fluoroquinolone, fusidic acid
NZ_CP053929.1 (951,842..953,055)	*mtrC*	9.18e-73	resistance-nodulation-cell division (RND) antibiotic efflux pump	macrolide, penam
WP_010460432.1	MexE	0	resistance-nodulation-cell division (RND) antibiotic efflux pump	phenicol, diaminopyrimidine, fluoroquinolone
WP_172791032.1	MexF	0	resistance-nodulation-cell division (RND) antibiotic efflux pump	phenicol, diaminopyrimidine, fluoroquinolone
WP_172791080.1	*Escherichia coli mdfA*	1.23e-80	major facilitator superfamily (MFS) antibiotic efflux pump	tetracycline, benzalkonium chloride, rhodamine
WP_083376511.1	*tetB(58)*	1.39e-9	major facilitator superfamily (MFS) antibiotic efflux pump	tetracycline antibiotic

WP_010457628.1	MexW	0	resistance-nodulation-cell division (RND) antibiotic efflux pump	disinfecting agents and intercalating dyes, phenicol, acridine dye, tetracycline, macrolide, fluoroquinolone
WP_010466790.1	SPM-1	2.71	SPM beta-lactamase	Carbapenem
WP_172791880.1	*bcr-1*	7.03e-30	major facilitator superfamily (MFS) antibiotic efflux pump	Bicyclomycin
WP_172791901.1	*bcrA*	6.56e-41	ATP-binding cassette (ABC) antibiotic efflux pump	peptide antibiotic
WP_083374963.1	AcrF	2.04	resistance-nodulation-cell division (RND) antibiotic efflux pump	penam, cephamycin, cephalosporin, fluoroquinolone
WP_010462522.1	OprM	0	resistance-nodulation-cell division (RND) antibiotic efflux pump	peptide antibiotic, macrolide diaminopyrimidine, penam, penem, cephamycin, sulfonamide, carbapenem, monobactam, cephalosporin, disinfecting agents and intercalating dyes, phenicol, tetracycline, aminoglycoside, aminocoumarin, acridine dye, fluoroquinolone

Table S2. Doubling time (hour) of *Pseudomonas* sp. B14-6

**Survival cells after treatment with 25 µg/ml gentamicin for 24 h**				
	Apramycin	Gentamicin	Kanamycin	Tobramycin
16× MIC	55.0 ± 6.1	27.8 ± 1.4	26.5 ± 1.7	38.8 ± 5.7
8× MIC	43.8 ± 5.0	29.3 ± 0.9	30.1 ± 2.9	31.5 ± 4.0
4× MIC	33.5 ± 1.9	33.3 ± 0.3	30.3 ± 1.3	27.5 ± 2.0
0× MIC	38.0 ± 1.0	35.6 ± 3.0	33.2 ± 1.0	36.5 ± 2.9
**Non-treated cells**				
	Apramycin	Gentamicin	Kanamycin	Tobramycin
16× MIC	No growth	No growth	No growth	No growth
8× MIC	No growth	No growth	No growth	No growth
4× MIC	No growth	No growth	No growth	No growth
0× MIC	1.9 ± 0.2	1.9 ± 0.3	1.9 ± 0.3	1.7 ± 0.2

Results represent mean ± S.D., n=3
